# Effect of acupuncture versus artificial tears for dry eye disease: A protocol for systematic review and meta-analysis

**DOI:** 10.1097/MD.0000000000021301

**Published:** 2020-07-24

**Authors:** Hongjuan Fu, Junxiang Wang, Feng Zhang, Yong Tang, Hao Zhou, Chao Wang

**Affiliations:** a School of Acupuncture-moxibustion and Tuina, Chengdu University of Traditional Chinese Medicine; b Sichuan Integrative Medicine Hospital, Chengdu, Sichuan; c School of Acupuncture-moxibustion and Tuina, Beijing University of Chinese Medicine, Beijing; d Acupuncture-moxibustion School of Affiliated Hospital of Chengdu University of Traditional Chinese Medicine, Chengdu, Sichuan, China.

**Keywords:** acupuncture, artificial tears, dry eye disease, meta-analysis, protocol, systematic review

## Abstract

**Background:**

The global prevalence of dry eye disease (DED) ranged from 5% to 50%, accompanied by the yearly increasing trend and younger onset. To date artificial tear serves as a mainstay therapy for DED management. It is noteworthy that, acupuncture has been accepted for treating DED with a time-honored history in China. However, no systematic review has been updated till now, which is focusing on comparing acupuncture vs. artificial tears for DED management.

**Methods:**

Eight databases will be searched with the language restrictions of English and Chinese from their inception to July 1, 2020. Randomized controlled trials comparing acupuncture versus artificial tears for DED treatment were enrolled. Identification, research inclusion, data extraction and assessment of the risk of bias were conducted independently by 2 or more reviewers. The primary outcomes were Schirmer I test and tear break-up time. We used Review Manager Software (v.5.3) for assessing the risk of bias and all statistical analyses.

**Results:**

Based on the evidence obtained, whether the effect of acupuncture was equal to or even better than that of artificial tear therapy in the treatment of DED would be elaborated.

**Conclusion:**

In summary, this review would provide a relatively convincing conclusion on whether acupuncture deserves to be recommended as an adjunct treatment for DED, so as to propose some significant insights for the doctors handling with DED.

**OSF Registration number:**

10.17605/OSF.IO/Z28M6.

## 1 Introduction

Dry eye disease (DED) refers to a group of tear film diseases caused by reduced tear production or tear film instability, normally accompanied by eye discomfort, visual symptoms, and inflammatory diseases of the ocular surface.^[1]^ According to a survey done by the Tear Film and Ocular Surface Society Dry Eye Workshop II Epidemiology subcommittee, the global prevalence of DED ranged from 5% to 50%.^[2]^ With the underlying factors, for example, air pollution, visual display terminals, and others, the incidence of DED seems to become popularly in the younger. Female gender, higher age, medications, Asian race and environmental exposure contribute to the increasing risk of DED.^[2,3]^ In fact, DED has became the perplex of the patients, who have to seek help from ophthalmologist.^[4]^

DED symptoms like ocular pain negatively impact the quality of life (QoL) of patients in reading, driving, and so on.^[5]^ In addition, the patients with DED is comparatively easier to be affected by psychiatric disorders, like depression.^[6]^ Unfortunately, DED can lead to decreased vision.^[7]^ Besides, it causes a substantial burden on patients and health-care systems due to the increases costs and reduced work productivity.^[8]^ In the US, the burden for managing DED is estimated to be $11,302 per patient, $3.84 billion to the healthcare system and $55.4 billion to the society overall.^[9]^ The prevalence and incidence have also increased exponentially in Spain,^[10]^ Germany^[11]^ and Asia,^[12]^ particularly in China.^[13]^ Therefore, a more safe, effective, and economic modality is well desired by both the doctors and patients.

At present, there is no fully curable modality for DED.^[2]^ Tear Film and Ocular Surface Society Dry Eye Workshop II and American Academy of Ophthalmology recommend artificial tears as the first-line treatment for patients with early-stage DED.^[1,2]^ Though some pharmacologic agents targeting the pathophysiology of DED show a slow therapeutic effect, the underlying drug-resistance cannot be ignored.^[14]^ In addition, a satisfaction survey revealed that many patients are unable to obtain effective symptom relief from anti-inflammatory drug, which are might along with some side effects.^[15]^ Only few physicians agree with the effectiveness of these treatments in relieving patients’ symptoms and improving their QoL.^[16]^ All the current situation ask a call for the effective and complementary treatments for DED.^[14,15]^

Acupuncture, as a significant branch of traditional Chinese medicine, has gained increasingly popularity in 183 countries and regions with its outstanding effects in treating several diseases, including DED.^[17–19]^ Interestingly, clinical study indicated that acupuncture could improve mid-term outcome compared to artificial tears.^[20]^ Based on the previous studies, we infer that acupuncture may be act as an effectively complementary therapy to the routine clinical treatment for DED.^[19,21]^

The tear film is composed of 3 layers: lipid, aqueous, and mucin. Any disorder of the related layers is close to the occurrence of DED.^[22]^ It is confirmed by studies that acupuncture can generate satisfying analgesic and anti-inflammatory effects.^[23,24]^ In addition, acupuncture can increase tear protein synthesis and secretion,^[21]^ also is good at in improving the tear meniscus dimensions for DED.^[25]^ Furthermore, Zhang et al have concluded that electroacupuncture can effectively regulate the conjunctival cytokine expressions.^[26]^ However, lacking of large samples of multi-center RCTs and strong evidences in the management of DED somewhat restrict the wide application of acupuncture clinically.

To date, here is only 1 systematic review comparing the efficacy of acupuncture and artificial tears in treating DED, and only 7 studies before 2012 were enrolled.^[27]^ Interestingly, acupuncture is not recommended as a complementary therapy in the latest management guidelines of DED.^[1]^ We, therefore, think, it is necessary to conduct a comprehensive review of the latest research on the effects of acupuncture vs. artificial tears on DED treatment, with the aim to explore whether acupuncture deserves to be recommended as an adjunct therapy for DED.

## 2 Methods

### 2.1 Study registration

This protocol was completed in accordance with the preferred reporting items for systematic reviews and meta-analyses protocols guidelines^[28]^ and registered in Open Science Framework with the registration number 10.17605/OSF.IO/Z28M6.

### 2.2 Eligibility criteria

#### 2.2.1 Type of study

Randomized controlled trials (RCTs) comparing acupuncture and artificial tears for the treatment of DED will be included.

#### 2.2.2 Type of participant

Patients meeting the diagnostic criteria for dry eye will be included regardless of age and gender. Pregnant women, or patients with secondary dry eye due to postoperative or Sjogren syndrome would be excluded.

#### 2.2.3 Type of intervention

Treatment group: acupuncture intervention, including electroacupuncture and milli-acupuncture, while control group: artificial tears intervention with different types. RCT studies involving different acupuncture programs, or other traditional Chinese medicine therapies (such as moxibustion), and combined intervention (acupuncture plus artificial tears) will be excluded.

#### 2.2.4 Type of outcome measure

According to the latest dry eye guidelines,^[1,22]^ Schirmer I test and Tear break-up time (BUT) will be used as the primary outcomes. Results of 10 mm or less for the Schirmer test and BUTs less than 10 seconds are generally considered abnormal. The secondary outcomes cover ocular surface disease index Questionnaire, ocular surface dry staining (including fluorescein or rose bengal), visual analogue scale (VAS), QoL questionnaire, adverse events, and effective rate.

### 2.3 Search methods for identification of studies

#### 2.3.1 Information sources

Two researchers (HJF and YT) independently search the following databases from their inception to 1 July 2020: PubMed, Embase, Web of Science, The Cochrane Library, Chinese National Knowledge Infrastructure, Wanfang Database, the Chongqing VIP Chinese Science and Technology Periodical Database, and Chinese Biomedical Literature Database. Ongoing trials will be searched from the NIH clinical registry ClinicalTrials. gov (https://www.clinicaltrials.gov/), the Chinese Clinical Trials Registry (http://www.chictr.org/en/) and the Acupuncture-Moxibustion Clinical Trials Registry (http://www.acmctr.org/index.aspx). In addition, the reference list of identified studies will be manually searched to find additional studies.

#### 2.3.2 Search

The search strategy is based on a combination of Medical Subject Headings (MeSH) and free texts. Search terms include “Xerophthalmia” “Dry Eye” “Dry Eye Syndromes” “Dry Eye Disease” “Keratoconjunctivitis Sicca” “Acupuncture” “Acupuncture therapy” “Electroacupuncture” “Artificial tears,” “Lubricant Eye Drops” and “Randomized controlled trial”. According to different languages, the search terms will be converted and used in different databases. The search strategy for The Cochrane Library is shown in Table 1.

**Table 1:**
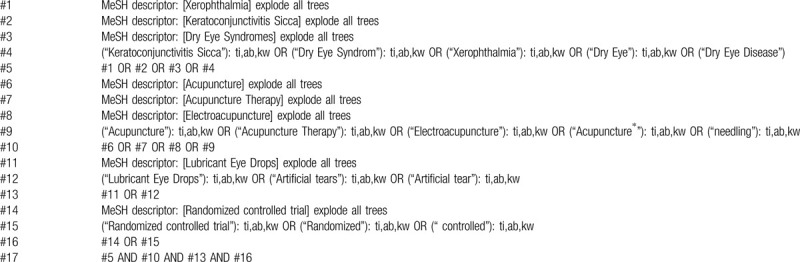
Search strategy for The Cochrane Library.

### 2.4 Data collection and analysis

#### 2.4.1 Study selection

**Figure 1:**
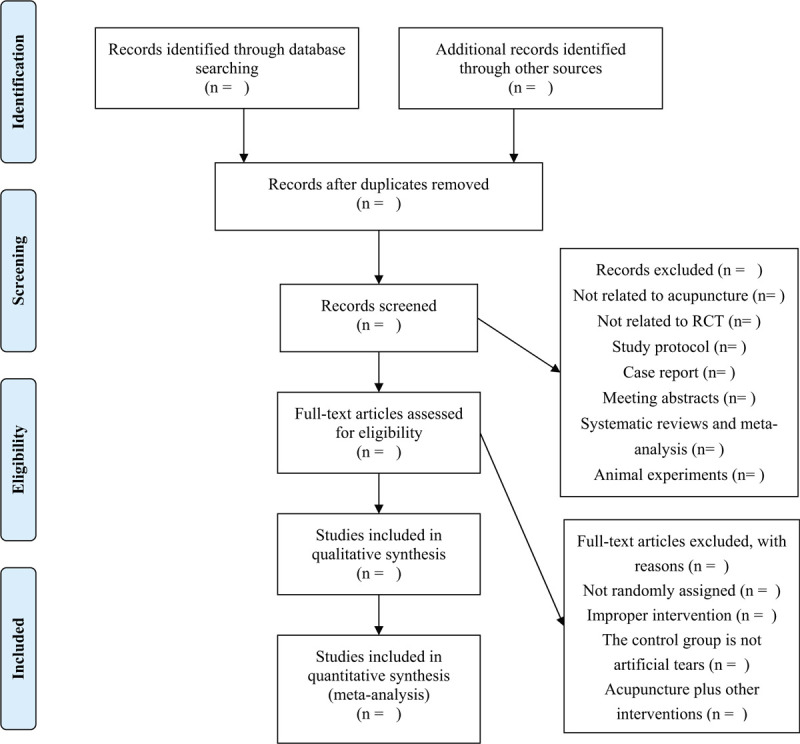
Flow chart of the study.

The study selection was performed independently by 2 reviewers (FZ and YT) and presented with the preferred reporting items for systematic reviews and meta-analyses flow chart (Fig. 1). After that, the results will be imported into EndNote X8 (Bld 10063) from the original databases, duplicate literatures will be removed. By reading the title and abstract, the reviewers excluded irrelevant literatures, non-RCTs, animal experiments, case reports, meeting abstracts, systematic reviews, and meta-analysis. Finally, read the full text to exclude RCTs that are not in accordance with the inclusion criteria. The exclusion reasons will be recorded in the Excel table. All inconsistencies will be resolved through discussion, and ultimately be decided by the third reviewer (HZ).

#### 2.4.2 Data collection process

Two reviewers (FZ and YT) independently check the eligibility of the included studies and then use the data extraction form to extract detailed information from each study. The extraction form mainly includes: citation information (title, first author, publication year, country), study design (random method, blind method, participants, interventions, intervention course), results (outcome measurement, adverse events) and conclusions. Cross-checking will be performed when data extraction is completed. Any disagreement during the data extraction process will be decided by the third researcher after discussion (HZ).

#### 2.4.3 Assessment of risk of bias (RoB)

The Cochrane Collaboration's tool for assessing RoB was used to assess the RoB in included studies.^[29]^ Two reviewers (HJF and JXW) independently input the detailed data of each study into Review Manager (v.5.3) to assess the RoB according to the following 7 aspects: (random sequence generation, allocation concealment, participant blinding, analyst blinding, data completeness of results, selective reports and other sources of bias). The results of the assessment will be cross-checked, and any inconsistencies will be arbitrated by the third reviewer after consultation (HZ).

#### 2.4.4 Summary measures

The mean difference or standard mean difference with 95% confidence interval will be analyzed for continuous data (eg, the Schirmer I test [SIT] value, BUT value), while risk ratio with 95% confidence interval for dichotomous data (eg, adverse events, effective rate).

#### 2.4.5 Unit of analysis issues

Individual patient data will not be involved in this review. Different units of the same outcome will be converted to international units before analysis.

#### 2.4.6 Dealing with missing data

Two reviewers (FZ and YT) will contact the corresponding author of the study by telephone or email to obtain the missing data. An intention-to-treat analysis and a sensitivity analysis will be performed if necessary.

#### 2.4.7 Assessment of heterogeneity

This protocol will use forest plot and *I*^2^ statistics to identify heterogeneity. There is a widespread belief that *I*^2^ values of 0 means no heterogeneity between studies; values of 25% low heterogeneity; values of 50% moderate heterogeneity; and values of 75% high heterogeneity.^[30]^ If there is significant heterogeneity between studies, we will explore the sources of heterogeneity and select a random effects model to merge the data.

#### 2.4.8 Synthesis of results

The fixed effects model will be used to synthesis the data when there is no heterogeneity between studies (*I*^2^ < 50%), while the random effects model for heterogeneity data (50% < *I*^2^ < 75%). Meta-analysis will not be performed when considerable heterogeneity is detected between the studies and a narrative summary will be presented.

#### 2.4.9 RoB across studies

As a rule of thumb, if more than 10 studies are included, tests for funnel plot asymmetry will be used to identify publication bias. We also further quantify the funnel plot asymmetry by using the Egger test, and define significant publication bias as a *P*-value < .1,^[31]^ rather than based on visual inspection of funnel plots alone.^[32]^

#### 2.4.10 Subgroup analysis

If conspicuous heterogeneity be found, we will interpret the heterogeneity by performing pre-planned subgroup analysis restricted to different types of artificial tears, Schirmer I test (local anesthesia or not) and acupuncture program (electro-acupuncture or milli-acupuncture).

#### 2.4.11 Sensitivity analysis

Sensitivity analysis can be done by changing some important factors affecting the results to determine the robustness of the conclusion. We also further evaluate the impact of a single study on the overall pooled estimate by removing 1 trial.

#### 2.4.12 Evidence quality evaluation

Since the reviewers did not comprehensively consider the overall quality of evidence when interpreting the pooled results, the following conclusions may be biased and misleading. Therefore, this protocol will use the grading of recommendations assessment, development, and evaluation system to assess the quality of evidence of the merged results. The quality of evidence will be specified to 4 grades: high, moderate, low, and very low quality.^[33]^

## 3 Discussion

The effective, safe, and economic therapeutic strategies are well needed by both doctors and patients due to the huge financial burden and current situation on DED management. Through this systematic review, we aim to provide a relatively convincing conclusion on whether acupuncture should be suggested as an adjunct treatment for DED. This research will be conducted in accordance with the 4 parts of identification, research inclusion, data extraction, and data synthesis. If it is necessary to modify this protocol, the date and details with reasons of the modification will be provided. There are also limitations in this review that only Chinese and English literatures will be included due to language barriers. Nonetheless, we believe that this research may provide reference to the clinical decision makers of DED.

## Author contributions

**Conceptualization:** Hongjuan Fu.

**Data curation:** Feng Zhang, Yong Tang, Hao Zhou.

**Formal analysis:** Junxiang Wang, Hongjuan Fu, Hao Zhou.

**Funding acquisition:** Chao Wang.

**Resources:** Chao Wang.

**Software:** Hongjuan Fu.

**Writing – original draft:** Hongjuan Fu.

**Writing – review & editing:** Junxiang Wang.
